# Supplementary material for: Renal survival and treatment of adult patients with Primary Focal Segmental glomerulosclerosis: A historical cohort study of the National Greek Registry
Source: PLoS One. 2024 Dec 18;19(12):e0315124. doi: 10.1371/journal.pone.0315124 (PMC11654980; doi:10.1371/journal.pone.0315124)
Supplement: S1 Table — (DOCX) [file pone.0315124.s001.docx]

**Supplementary Table 1.** Baseline characteristics by ESRD status.

| Characteristics | ESRD  N=88 | No ESRD  N=302 | p-value |
| --- | --- | --- | --- |
|  | N (%), Median (IQR), Mean (SD) | |  |
| Age (years)^1^ | 47 (±17) | 45 (±15) | 0.37 |
| Sex (Males) | 57 (65%) | 194 (64%) | 0.90 |
| BMI (kg/m^2^)^2^ | 26 (22-31) | 27 (24-32) | 0.64 |
| Proteinuria (g/d)^2^ | 4 (2.37-6.1) | 3.8 (2-6) | 0.21 |
| Albumin (g/dL)^2^ | 3.55 (2.6-4.1) | 3.6 (2.8-4.1) | 0.43 |
| Baseline eGFR (ml/min per 1.73 m^2^)^2^ | **43 (30-71.5)** | **72 (51-96)** | **<0.001** |
| Hypertension | **67 (76%)** | **200 (66%)** | **0.02** |
| Immunosuppression | 58 (66%) | 194 (64%) | 0.21 |
| ACEi or ARB therapy | 55 (62%) | 229 (76%) | 0.14 |
| Remission | | | **<0.001** |
| - CR | **4 (5%)** | **127 (42%)** |  |
| - PR | **21 (24%)** | **129 (43%)** |  |
| - NR | **47 (53%)** | **38 (13%)** |  |
| Relapse | **24 (93%)** | **109 (46%)** | **<0.001** |
| Number of Relapses^2^ | **1 (1-2)** | **0 (0-1)** | **<0.001** |

1: Mean (SD), 2: Median (IQR)

BMI: Body Mass Index, ACEi: Angiotensin-converting enzyme inhibitors, ARB: Angiotensin Receptor Blockers, eGFR: estimated Glomerular Filtration Rate, CR: Complete Remission, PR: Partial Remission, NR: No Remission, ESRD: End Stage Renal Disease
