# Supplementary material for: Renal survival and treatment of adult patients with Primary Focal Segmental glomerulosclerosis: A historical cohort study of the National Greek Registry
Source: PLoS One. 2024 Dec 18;19(12):e0315124. doi: 10.1371/journal.pone.0315124 (PMC11654980; doi:10.1371/journal.pone.0315124)
Supplement: S2 Table — (DOCX) [file pone.0315124.s002.docx]

**Supplementary Table 2**. Clinical and laboratory parameters by immunosuppressive regimen.

| Immunosuppressive Regimen | Glucocorticoids only  Ν= 135 | Cyclosporine ± glucocorticoids  Ν= 126 | p-value |
| --- | --- | --- | --- |
| Age (years)^1^ | 43 (±15) | 45 (±15) | 0.14 |
| Sex (Males) | 90 (67%) | 78 (61%) | 0.42 |
| BMI (kg/m^2^)^2^ | 25.3 (22.9-30.9) | 28 (23.9-32) | 0.12 |
| Proteinuria (g/d)^2^ | 4.8 (3.4-7.5) | 5.3 (3.9-7.2) | 0.22 |
| Albumin (g/dL)^2^ | 3 (2.3-4) | 3 (2.4-3.8) | 0.38 |
| Baseline eGFR (ml/min per 1.73 m^2^)^2^ | 65 (35-98) | 71 (52.6-91) | 0.22 |
| Hypertension | 79 (59%) | 88 (70%) | 0.07 |
| ACEi or ARB therapy | **80 (60%)** | **106 (85%)** | **<0.001** |
| Remission |  |  | 0.10 |
| - CR | 55 (44%) | 47 (38%) |  |
| - PR | 43 (34%) | 59 (47%) |  |
| - NR | 27 (22%) | 19 (15%) |  |
| ESRD | 27 (25%) | 23 (22%) | 0.51 |
| Relapse | 49 (57%) | 66 (68%) | 0.12 |
| Number of Relapses^2^ | 1 (0-1) | 1 (0-2) | 0.11 |

1: Mean (SD), 2: Median (IQR)

BMI: Body Mass Index, ACEi: Angiotensin-converting enzyme inhibitors, ARB: Angiotensin Receptor Blockers, eGFR: estimated Glomerular Filtration Rate, CR: Complete Remission, PR: Partial Remission, NR: No Remission, ESRD: End Stage Renal Disease
