# Supplementary material for: Renal survival and treatment of adult patients with Primary Focal Segmental glomerulosclerosis: A historical cohort study of the National Greek Registry
Source: PLoS One. 2024 Dec 18;19(12):e0315124. doi: 10.1371/journal.pone.0315124 (PMC11654980; doi:10.1371/journal.pone.0315124)
Supplement: S1 Fig — Survival from renal failure in patients with primary FSGS, according to the time of the diagnosis. (DOCX) [file pone.0315124.s003.docx]

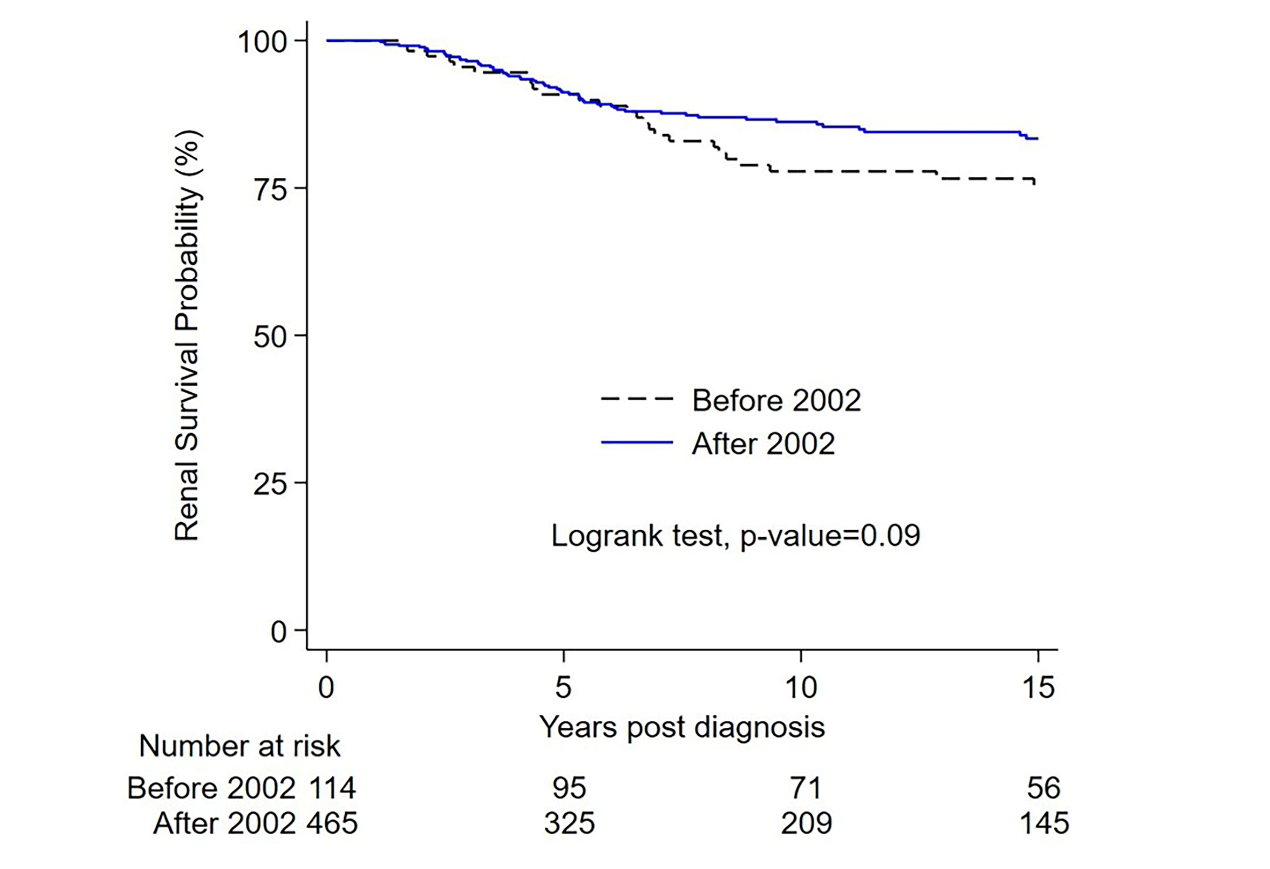


**Supplementary Figure 1.** Kaplan-Meier Curves. Survival from renal failure in patients with primary FSGS, according to the time of the diagnosis.
